# Salinity in Autumn-Winter Season and Fruit Quality of Tomato Landraces

**DOI:** 10.3389/fpls.2019.01078

**Published:** 2019-09-24

**Authors:** Tommaso Michele Moles, Rita de Brito Francisco, Lorenzo Mariotti, Antonio Pompeiano, Antonio Lupini, Luca Incrocci, Giulia Carmassi, Andrea Scartazza, Laura Pistelli, Lorenzo Guglielminetti, Alberto Pardossi, Francesco Sunseri, Stefan Hörtensteiner, Diana Santelia

**Affiliations:** ^1^Department of Agriculture, Food and Environment, University of Pisa, Pisa, Italy; ^2^Institute of Integrative Biology, ETH Zürich, Zürich, Switzerland; ^3^Department of Plant and Microbial Biology, University of Zürich, Zürich, Switzerland; ^4^International Clinical Research Centre, St. Anne’s University Hospital, Brno, Czechia; ^5^Central European Institute of Technology, Brno University of Technology, Brno, Czechia; ^6^Department of Agraria, University Mediterranea of Reggio Calabria, Reggio Calabria, Italy; ^7^Institute of Research on Terrestrial Ecosystems, National Research Council, Pisa, Italy

**Keywords:** tomato, landraces, off-season, salinity, fruit quality, metabolites

## Abstract

Tomato landraces, originated by adaptive responses to local habitats, are considered a valuable resource for many traits of agronomic interest, including fruit nutritional quality. Primary and secondary metabolites are essential determinants of fruit organoleptic quality, and some of them, such as carotenoids and phenolics, have been associated with beneficial proprieties for human health. Landraces’ fruit taste and flavour are often preferred by consumers compared to the commercial varieties’ ones. In an autumn-winter greenhouse hydroponic experiment, the response of three Southern-Italy tomato landraces (Ciettaicale, Linosa and Corleone) and one commercial cultivar (UC-82B) to different concentrations of sodium chloride (0 mM, 60 mM or 120 mM NaCl) were evaluated. At harvest, no losses in marketable yield were noticed in any of the tested genotypes. However, under salt stress, fresh fruit yield as well as fruit calcium concentration were higher affected in the commercial cultivar than in the landraces. Furthermore, UC-82B showed a trend of decreasing lycopene and total antioxidant capacity with increasing salt concentration, whereas no changes in these parameters were observed in the landraces under 60 mM NaCl. Landraces under 120 mM NaCl accumulated more fructose and glucose in the fruits, while salt did not affect hexoses levels in UC-82B. Ultra-performance liquid chromatography–tandem mass spectrometry analysis revealed differential accumulation of glycoalkaloids, phenolic acids, flavonoids and their derivatives in the fruits of all genotypes under stress. Overall, the investigated Italian landraces showed a different behaviour compared to the commercial variety UC-82B under moderate salinity stress, showing a tolerable compromise between yield and quality attributes. Our results point to the feasible use of tomato landraces as a target to select interesting genetic traits to improve fruit quality under stress conditions.

## Introduction

Tomato is the most consumed berry fruit worldwide as well as one of the most important constituents of the Mediterranean diet representing a key source of minerals, vitamins and antioxidants (Canene-Adams et al., 2005). Fruit quality is affected by environmental conditions, such as seasonal changes, the occurrence of biotic/abiotic stress and agronomic practices (water management and fertilizer supply), as well as genetic factors (Poiroux-Gonord et al., 2010), but their mechanism of action is not completely clear. To enhance health-related compounds, different agronomic strategies have been applied, namely grafting (Sánchez-Rodríguez et al., 2012; Casals et al., 2018) or controlled water management techniques (Barbagallo et al., 2008).

Salinity induces changes in physiology and metabolism that affect the final crop yield (Pompeiano et al., 2016). Tomato is generally considered a moderately salt-tolerant crop, often cultivated in areas polluted by salinization of aquifers and consequent use of saline water for irrigation (Santa-Cruz et al., 2002). Salinity can positively modulate tomato fruit metabolism and improves the sensorial/nutritional value of the production (D’Amico et al., 2003). Salinity can increase the total soluble content (°Brix) and the titratable acidity, two important parameters influencing the quality of tomato fruits. Moreover, a high salt concentration in irrigation water generally stimulates the defence system of the plant, thereby leading to accumulation of secondary metabolites in different tissues. One common feature of plant secondary compound classes, such as carotenoids, polyphenols and terpenoids, is reactive oxygen species (ROS) scavenging activity (Ndhlala et al., 2010). Due to their strong antioxidant activity, these bioactive metabolites have been recognized as beneficial players against human cardiovascular and chronic degenerative diseases (Borguini and da Silva Torres, 2009) and tumours (Barone et al., 2018). High salinity can accelerate lycopene biosynthesis in hydroponically-grown tomato plants (Wu and Kubota, 2008). Several water management techniques applying controlled and moderate drought/salt stress in the pre-harvesting period of tomato fruits have been implemented to maintain a sufficient yield and also to produce fruits with improved nutritional level (Kubota et al., 2012).

The diurnal and seasonal changes in light intensity, vapour pressure and temperature can also explain the differences observed between seasonal experiments (spring-summer vs autumn-winter) and cultivation systems (open-field vs greenhouse) (Incerti et al., 2009; Asensio et al., 2019). Yield and quality of tomato fruits from off-season greenhouse cultivation are often reduced compared to open-field production (Hu et al., 2006). This effect depends on the association of the different climatic conditions with the covering materials used in the protected environment—that can deplete the intensity and the quality of the light spectrum inside the greenhouse (Toor et al., 2006; Gent, 2007; Mariz-Ponte et al., 2019). Light quality and intensity are indeed major constraints influencing quality parameters in tomato fruit (Slimestad and Verheul, 2005). Generally, °Brix and titratable acidity are positively correlated with increasing light intensity (Claussen et al., 2006) and temperature (Adams et al., 2001). High light intensity and the modulation of UV-B in the light spectra enhance flavonoid accumulation in tomato fruit tissues. Low night temperature, which often occurs in non-heated greenhouses during winter, drastically affects plant growth and crop yield (Jing et al., 2016).

The genetic background is a critical factor that can significantly influence fruit quality (Steward et al., 2000; Vallverdú-Queralt et al., 2011). In the present work the response to salt stress of long-storage tomato landraces (or traditional varieties) were assessed. In particular, the effects of moderate and high concentrations of sodium chloride (60 and 120 mM NaCl) on yield and quality-related fruit metabolites were evaluated on three Italian tomato landraces from different geographic origin (Ciettaicale, Corleone and Linosa). These landraces are traditionally used to prepare fresh sauce or dried fruits stored in olive oil. A tomato ancient variety (UC-82B) was included in the experiment as commercial control. According to our preliminary screening of a tomato landrace collection, the local accessions selected for this study represented promising candidates for traits related to abiotic stress tolerance, which are notably found in other Mediterranean tomato landraces (Galmes et al., 2013; Patanè et al., 2016). Indeed, compared with other processing tomato genotypes, Ciettaicale previously showed an interesting tolerance profile to salt and drought stress at vegetative stage (Moles et al., 2016; Moles et al., 2018), whereas Linosa exhibited a high nitrogen use efficiency (Abenavoli et al., 2016; Lupini et al., 2017). These promising findings prompted to carry out experiments in autumn-winter off-season climatic conditions, which allowed us to adopt a hydroponic irrigation system with different salt concentrations (60 and 120 mM NaCl can be considered as 10% and 20% seawater dilutions, respectively) that normally are considered high for a moderate salt-sensitive crop such as tomato (Cuartero et al., 2006). This information is essential for selecting potential metabolic traits to be used as biomarkers on which to focus for new breeding strategies.

## Materials and Methods

### Plant Material and Growth Condition

Three Southern Italy tomato landraces and a standard tomato variety were used as genetic material in this study. Among the landraces, Ciettaicale (from Basilicata region) and Linosa (from Pelagic Islands, Sicily) belong to the category of tomatoes with indeterminate growth habit and with pear/globose fruits, whereas Corleone (from Sicily region) is an indeterminate tomato type with flattened/ribbed fruits. The commercial variety UC-82B (supplied by the Tomato Genetics Resource Center, Department of Plant Sciences, University of California-Davis, CA, USA) belongs to the category of determinate tomatoes with pear/globose fruits. Plants were grown in rockwool cube (Rockwool B.V., The Netherlands), in an open nutrient solution system at a plant density of approximately 3 m^−2^ in a glasshouse at the University of Pisa (Italy) from September 2016 to February 2017. The plants (16 individuals for each of the 4 genotypes for each of 3 experimental conditions, divided in eight pairs which were arranged in a randomized block design on the glasshouse benches, for a total of 192 plants) were grown vertically trimming the plant below the fourth fruit truss. Climatic parameters were continuously monitored by means of a weather station located inside the glasshouse. The mean air temperature and relative humidity were 17.3°C and 74.6%, respectively (T_min_ = 11.4°C and T_max_ = 27.7°C; RH_min_ = 50.2% and RH_max_ = 96.7%). Mean values of daily inside global radiation was 3.44 MJm^−2^ (GR_min_ = 1.02 MJm^−2^ and GR_max_ = 10.30 MJm^−2^). Two salinity levels of nutrient solution were used with electrical conductivities (EC) of 8.3 and 14.6 mS cm^−1^, which corresponded roughly to 60 and 120 mM NaCl, respectively. The concentration of nutrient solution was as reported by Incerti et al. (2007). Salt stress was applied 3 weeks after planting; the process was stepped up in roughly 2.1 mS cm^−1^ (20 mM NaCl) daily increments to avoid osmotic shock. Irrigation was controlled by a timer that opened the irrigation lines for 1 min up to 12 times per day, depending on growing stage and environmental conditions.

### Biometrical Measurements, Fruit Yield and Sampling

Fresh fruit yield (FW) was determined based on total fruits picked two times per week in February from all the three trusses of each plant and genotype under different experimental treatments. The red-ripe fruits from the second truss (which consistence was evaluated by penetrometer) were separately collected early in the morning, weighted and randomly grouped in six biological replicates (15-20 whole fruits composed a single biological replicate) to be ground altogether at 4°C (homogenate) and then used for laboratory determinations. An aliquot of the homogenate was added to the remaining harvested material and stored at 80°C for 2 weeks to record fruit dry weight (DW). Aliquots of the homogenate were separately stored and placed at 80°C for 1 week for cation quantifications; additional aliquots were used to determine °Brix and titratable acidity (Beckles, 2012); finally, other samples were collected in tubes and stored at -80°C for further metabolic analyses (per biological replicate, four technical replicates were analysed). At the end of experiment, leaf area was estimated using a digital planimeter; then, shoot organs (leaves and stems) were weighted (shoot FW) and put in 80°C for 2 weeks to record the dry weight (shoot DW).

### Total Soluble Sugar Measurements

Glucose, fructose and sucrose were extracted from tomato fruit homogenate aliquots according to the protocol described in Hostettler et al. (2011), and then quantified enzymatically according to Thalmann et al. (2016).

### Cation Determination

Fruit dried samples were powdered and mineralized (60 min at 220°C) using a solution of HNO_3_:HClO_4_ (2.5:1, v/v). Sodium (Na^+^), potassium (K^+^) and calcium (Ca^2+^) were determined using an atomic absorption spectrometer (Varian AA 24FS, Australia).

### Lycopene Determination

Lycopene content was assayed according to Giovannetti et al. (2012). Briefly, an aliquot of tomato fruit homogenate was extracted in a solution of acetone:ethanol:hexane (1:2:1, v/v/v) and agitated on an orbital shaker for 15 min. Then one volume of distilled water was added, followed by 5 min agitation. After centrifugation, the hexane phase was measured at 503 nm, blanked with pure hexane.

### Total Flavonoids, Total Phenols and Total Antioxidant Activity Contents

Tomato fruit homogenates were mixed in 70% (v/v) methanol and agitated overnight at 4°C in the dark. After incubation, the extracts were centrifuged at 12000 x g for 15 min at 4°C and the supernatants were utilized for the analyses indicated below. Total soluble phenols content (TPHE) and total flavonoids content (TFL) were assayed using the respective protocols reported in Caser et al. (2016). Total antioxidant capacity (TAC) was determined by the 2,2-diphenyl-1-picrylhydrazyl (DPPH) assay as previously reported in Moles et al. (2016), with some modifications. Briefly, an aliquot of the methanolic fruit extract was added to 0.1 mM methanolic DPPH solution. After 30 min of incubation at room temperature in the dark, absorbance was measured at 515 nm, and the results were expressed as µmol of Trolox per gram of plant material on dry basis.

### Metabolite Profiling

Tomato fruit homogenate (100 mg) was extracted with 100% methanol. The samples were ground using a mixer mill with 1.25–1.65 mm glass beads for 1.5 min at 30 Hz, centrifuged at 15,000 × g at 4°C and the supernatants collected. All samples were de-salinized over a silica-based classic cartridge (WAT051910, Waters) according to the manufacturer instructions. The eluted samples were concentrated using a Savant SpeedVac concentrator (Thermo Fisher Scientific) at 42°C. Prior to ultra-performance liquid chromatography–tandem mass spectrometry (UPLC-MS/MS) analysis the samples were re-suspended in 80% methanol, 0.1% formic acid. After sonication for 5 min, the samples were centrifuged at 15,000 × g, at 4°C for 5 min, and transferred to liquid chromatography vials. Samples were analysed on a UPLC (Dionex UltiMate 3000, Thermo Fisher Scientific) coupled to a Bruker compact electrospray ionisation (ESI)-quadrupole-time-of-flight tandem-mass spectrometer (Bruker Daltonics). The UPLC separation was performed at 28°C with a C18 reverse-phase column (ACQUITY UPLC™ BEH C18, 1.7 µm, 2.1 × 150 mm, Waters) using the following gradient of solvent B [acetonitrile with 0.1% (v/v) formic acid] and solvent A [water with 0.1% (v/v) formic acid]: 0–0.5 min, 5% B; 0.5–12 min, 5–100% B; 12–14 min, 100% B; 14–16 min, 100–5% B. The flow rate was set to 0.3 mL min^−1^ and 5 µl of each sample was injected. The ESI source was operated in positive mode and parameters were set as follows: gas temperature, 220°C; drying gas, 9 L  min^−1^; nebuliser, 2.2 bar; capillary voltage, 4,500 V; end plate offset, 500 V. The instrument was set to acquire an m/z range of 50–1,300. Conditions for MS/MS were set as described by Christ et al. (2016). All data were recalibrated internally using pre-run injection of 10 mM sodium hydroxide in 0.2% formic acid, 49.8% water, 50% isopropanol (v/v/v). DataAnalysis v.4.2 and TargetAnalysis v.1.3 softwares (Bruker Daltonics) were used to analyse the data. Metabolites were identified and annotated by comparison with MS and MS/MS data spectra either generated by authentic reference standards or deposited in the literature and databases, such as PubChem and MoTo (Moço et al., 2006).

### Statistical Analysis

A randomized block design as previously described was performed. Data were subjected to two-way analysis of variance (ANOVA) and the mean values were compared using Duncan’s test (P < 0.05) to check the significant differences. For the metabolomics data, in order to examine the differences between the treatments, the percentage contribution of each compound to the average dissimilarity between the aforementioned factors was calculated using similarity percentage analysis (SIMPER) (Vaníčková et al., 2015). A cut-off was imposed where ∑δi% reached 70%. Also, the differences in the chemical composition and extra characteristics of the samples from the study were analysed by principal component analysis (PCA). Prior to PCA, peak areas were subjected to logarithmic transformation; intraspecific scaling was performed by dividing each treatment/experimental condition score by its standard deviation; the data were centred by treatment scores. In PCA analyses, hierarchical clustering based on Pearson correlation showed that treatments with similar chemical profiles cluster together. All computations were performed with R 3.5.3 language and environment (R Core Team, 2019) and the R packages FactoMineR (Le et al., 2008) and vegan (Oksanen et al., 2019).

## Results

We assessed the effects of moderate and high salt treatments (60 and 120 mM NaCl) applied during an off-season greenhouse experiment, thereby evaluating yield and quality-related fruit metabolites in three Italian tomato landraces (Ciettaicale, Corleone and Linosa) and in a commercial variety (UC-82B). Analysis of all the biometric and metabolic traits revealed a significant (P < 0.05) genotype × treatment interaction. Following that, subsequent data were presented in treatment combinations.

### Biometrical Measurements and Fruit Yield

To investigate whether (or how) salinity stress impacted on leaf growth, we measured leaf area and epigeal biomass at the end of the experiment. In the landraces leaf area did not decrease upon treatment with 60 mM NaCl (**Supplementary Figure 1A**), while a significant reduction in this parameter was recorded under 120 mM NaCl, particularly in Ciettaicale (approximately 46% smaller than its control). By contrast, the commercial variety UC-82B decreased leaf area about 30% and 60% under 60 and 120 mM NaCl, respectively. However, a common reduction in the vegetative biomass (shoot FW and DW) was recorded in all the tomato genotypes comparing to their respective controls (**Supplementary Figures 1B, C**).

**Figure 1:**
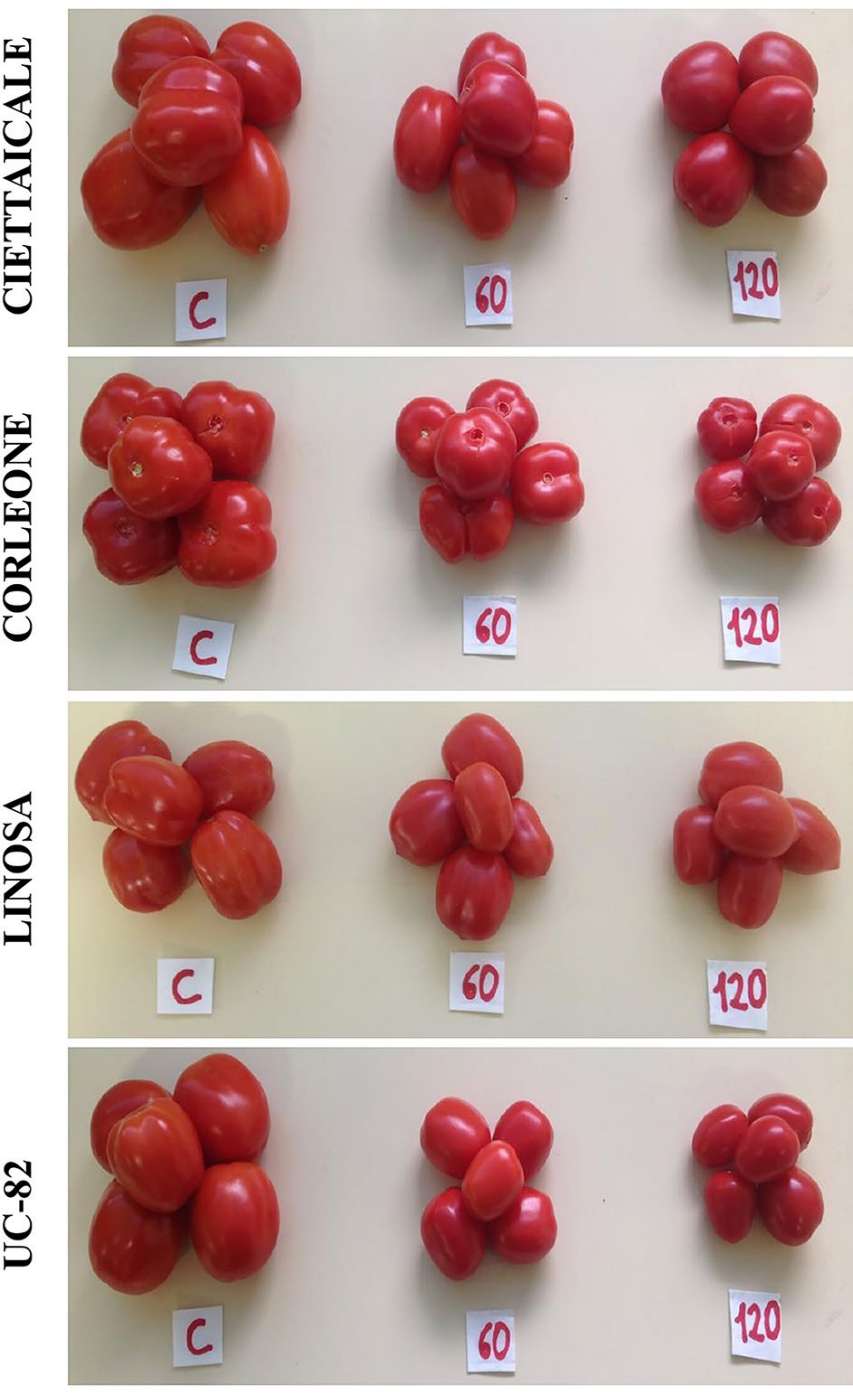
Effect of different salt concentrations (60 mM and 120 mM NaCl) on fruit size of three tomato landraces (Ciettaicale, Corleone and Linosa) and a commercial variety (UC-82B) compared to respective control condition (C).

**Figure 2:**
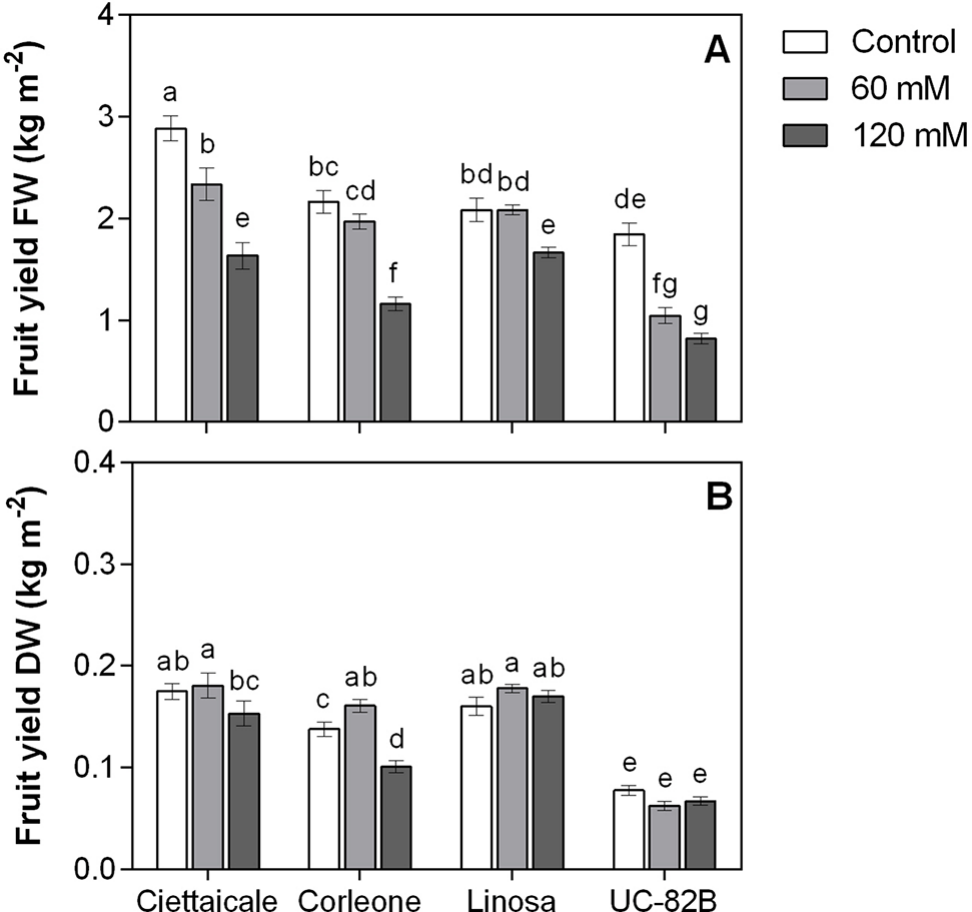
Effect of different salt concentrations (60 mM and 120 mM NaCl) on fruit yield of three tomato landraces (Ciettaicale, Corleone and Linosa) and a commercial variety (UC-82B). **(A)** Yield fresh weight (FW) and **(B)** yield dry weight (DW). Error bars represent the standard error of the mean (n = 16). Bars with same letters are not statistically different from one another according to Duncan’s test (P < 0.05).

Although salinity treatments differently affected fruit size (**Figure 1** and **Table 1**), the number of fruits was unaffected (**Table 1**). Ciettaicale and UC-82B already reduced fruit yield FW under 60 mM NaCl (**Figure 2A**). By contrast, Corleone and Linosa decreased fruit yield FW only under 120 mM NaCl by 47% and 20%, respectively. Moreover, Ciettaicale, Linosa and UC-82B did not exhibit significant differences when considering fruit yield DW (**Figure 2B**), except Corleone which significantly increased fruit yield DW under 60 mM NaCl (about 14%) and decreased under 120 mM Na Cl by 27% compared to the control.

**Table 1:** Effect of different salt concentrations (60 mM and 120 mM NaCl) on number of fruits, single fruit fresh weight (FW), fruit total soluble solids (°Brix) and titratable acidity (TA) of three tomato landraces (Ciettaicale, Corleone and Linosa) and a commercial variety (UC-82B).

Genotype	[NaCl]	n° fruits plant^-1^	Single fruit FW (g plant^-1^)	°Brix	TA
Ciettaicale	Control	18.25 ± 0.74 b	51.12 ± 3.66 a	3,84 ± 0.25 e	0,26 ± 0.01 d
	60 mM	17.94 ± 0.87 b	41.29 ± 1.69 cd	4,94 ± 0.15 cd	0,29 ± 0.01 c
	120 mM	18,25 ± 0.63 b	32.47 ± 1.44 e	6,28 ± 0.13 b	0,33 ± 0.01 b
Corleone	Control	14.81 ± 0.86 cd	48.57 ± 0.84 ab	4,10 ± 0.10 e	0,29 ± 0.01 ce
	60 mM	16.81 ± 0.78 bc	39.24 ± 0.97 d	4,60 ± 0.18 d	0,30 ± 0.01 c
	120 mM	14.38 ± 0.64 d	25.83 ± 1.04 fg	6,44 ± 0.13 b	0,39 ± 0.01 a
Linosa	Control	24.44 ± 0.89 a	29.32 ± 0.49 eg	5,04 ± 0.11 c	0,27 ± 0.01 cd
	60 mM	23.63 ± 0.69 a	29.99 ± 0.71 ef	6,02 ± 0.12 b	0,33 ± 0.01 b
	120 mM	23.13 ± 0.64 a	24.72 ± 0.71 gh	7,14 ± 0.12 a	0,29 ± 0.01 c
UC-82B	Control	13.00 ± 0.82 d	44.43 ± 2.32 bc	2,50 ± 0.16 f	0,17 ± 0.01 e
	60 mM	13.50 ± 0.64 d	26.91 ± 1.10 fg	4,14 ± 0.12 e	0,25 ± 0.01 d
	120 mM	13.06 ± 0.61 d	21.09 ± 1.03 h	4,70 ± 0.07 cd	0,36 ± 0.02 a

Data are means *±* SE of 16 replicates for the biometrical parameters and of 6 replicates for fruit quality estimations, respectively. Means within a column followed by the same letter are not significantly different based on Duncan’s test (P < 0.05).

### Soluble Sugars Content

**Figure 3:**
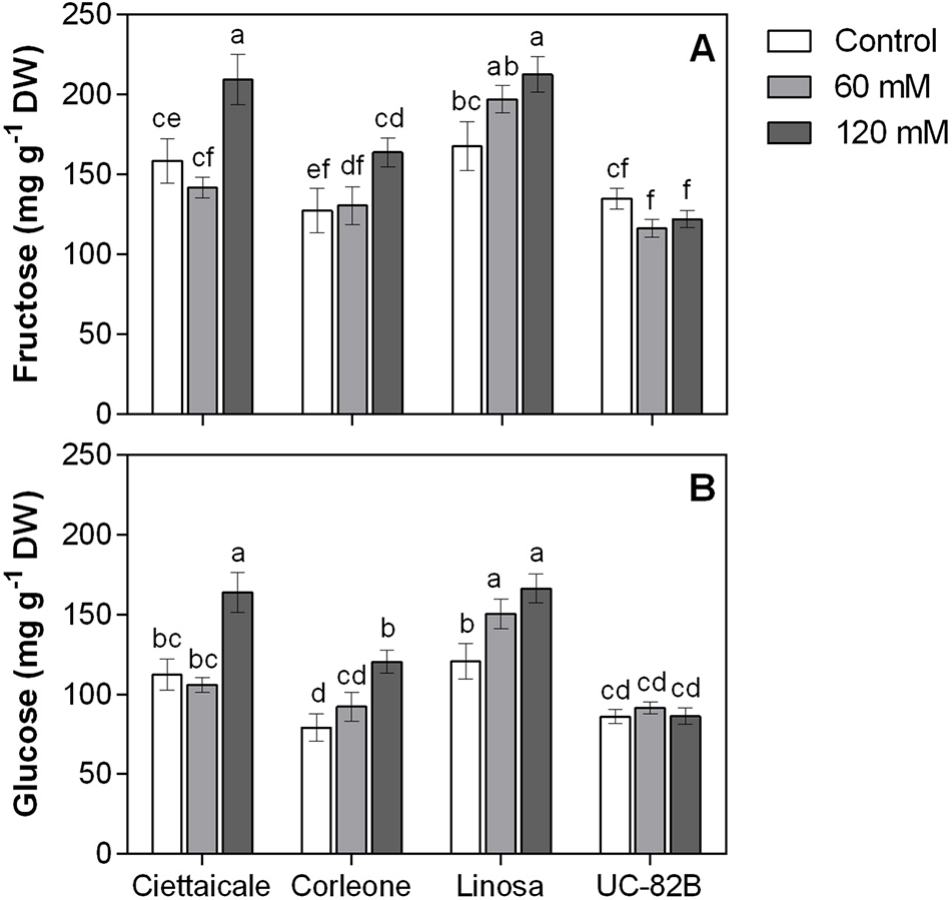
Effect of different salt concentrations (60 mM and 120 mM NaCl) on fruit hexose contents of three tomato landraces (Ciettaicale, Corleone and Linosa) and a commercial variety (UC-82B). **(A)** Fructose content and **(B)** glucose content. Error bars represent the standard error of the mean (n = 6). Bars with same letters are not statistically different from one another according to Duncan’s test (P < 0.05).

In all genotypes, 120 mM NaCl treatment caused significant increases in both fruit °Brix and titratable acidity (**Table 1**). We did not detect sucrose in fruit samples, but different trends in glucose and fructose contents among tomato genotypes were observed (**Figure 3**). Under 120 mM NaCl, landraces had increased glucose and fructose contents, while no significant changes in glucose and fructose levels were observed in the commercial variety under the salt treatment.

### Cation Content

Salt treatments differently affected the fruit contents of Na^+^, K^+^ and Ca^2+^, depending on the genotype (**Table 2**). Ciettaicale showed the highest fold increase, reaching 3.4 fold more under 60 mM NaCl and 5.3 fold more under 120 mM NaCl compared to the control. Under 120 mM NaCl, the lowest Na^+^ concentration was found in Linosa fruits (2.5 fold more than the control), while the highest Na^+^ level was recorded in UC-82B (4 fold more than the control). Notably, Ciettaicale accumulated more K^+^ under both salinity treatments than in control, and more K^+^ was also found in Linosa under 60 mM NaCl. However, 120 mM salt induced a decrease of K^+^ concentration in Corleone and UC-82B. Commercial variety fruits showed reduced Ca^2+^ content already at 60 mM NaCl, while Linosa had less Ca^2+^ only under 120 mM compared to respective controls. Overall, Linosa maintained higher K^+^/Na^+^ and Ca^2+^/Na^+^ ratios at 60 mM and 120 mM NaCl compared to the other genotypes, while Ciettaicale and UC-82B more markedly decreased Ca^2+^/Na^+^ ratio with increasing salinity level.

**Table 2:** Effect of different salt concentrations (60 and 120 mM NaCl) on fruit cation contents of three tomato landraces (Ciettaicale, Corleone and Linosa) and a commercial variety (UC-82B). Data are means ± SE of six replicates.

Genotype^+^	[NaCl]	Na^+^ (g kg^-1^ DW)	K^+^ (g kg^-1^ DW)	Ca^2+^ (g kg^-1^ DW)	K^+^/Na^+^	Ca^2+^/Na^+^
Ciettaicale	Control	0.62 ± 0.03 f	30.03 ± 0.94 f	1.70 ± 0.09 cd	48.96 ± 2.92 a	2.76 ± 0.20 a
	60 mM	2.12 ± 0.22 cd	36.47 ± 2.26 bd	1.61 ± 0.13 cd	17.38 ± 1.03 d	0.77 ± 0.06 e
	120 mM	3.31 ± 0.31 b	36.29 ± 1.22 bd	1.71 ± 0.09 bd	11.18 ± 1.27 ef	0.52 ± 0.04 eg
Corleone	Control	0.95 ± 0.09 f	39.21 ± 0.81 ab	1.57 ± 0.14 cd	41.94 ± 3.35 b	1.65 ± 0.04 e
	60 mM	1.99 ± 0.02 de	38.22 ± 0.89 ac	1.56 ± 0.12 cd	19.20 ± 0.30 d	0.79 ± 0.06 c
	120 mM	3.48 ± 0.11 b	34.38 ± 0.65 de	1.45 ± 0.04 d	9.90 ± 0.43 ef	0.42 ± 0.01 fg
Linosa	Control	0.89 ± 0.07 f	36.93 ± 1.39 bd	1.78 ± 0.13 ac	42.10 ± 3.68 b	2.02 ± 0.20 b
	60 mM	1.67 ± 0.06 e	41.29 ± 1.04 a	2.02 ± 0.03 a	24.79 ± 1.23 c	1.21 ± 0.05 d
	120 mM	2.26 ± 0.61 cd	35.38 ± 0.47 ce	1.49 ± 0.03 cd	15.73 ± 0.53 de	0.66 ± 0.02 ef
UC-82B	Control	1.00 ± 0.02 f	37.46 ± 0.70 bd	1.99 ± 0.10 b	37.50 ± 1.21 b	2.00 ± 0.12 b
	60 mM	2.50 ± 0.20 c	35.83 ± 1.06 bd	1.16 ± 0.06 e	14.61 ± 1.69 de	0.47 ± 0.03 fg
	120 mM	3.98 ± 0.14 a	32.00 ± 0.94 ef	1.10 ± 0.06 e	8.05 ± 0.04 f	0.28 ± 0.01 g

Means within a column followed by the same letter are not significantly different based on Duncan’s test (P < 0.05).

### Lycopene

**Figure 4:**
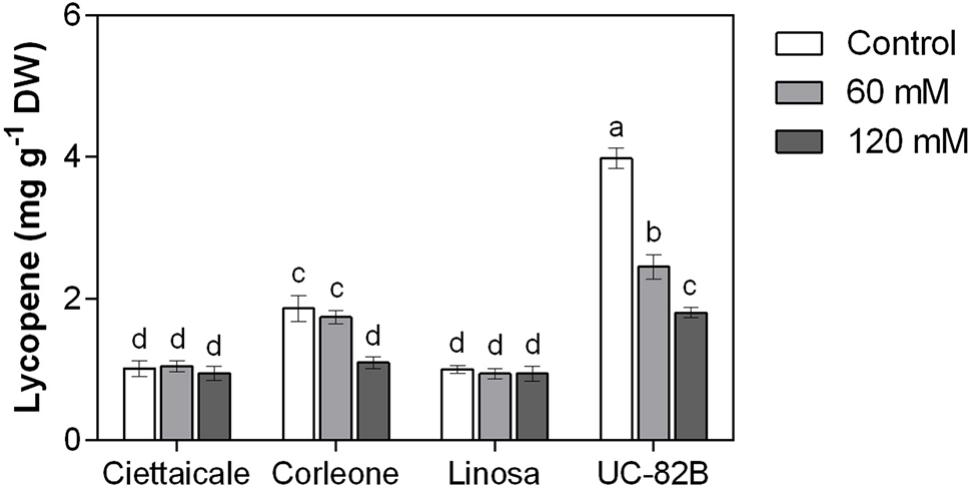
Effect of different salt concentrations (60 mM and 120 mM NaCl) on fruit lycopene content of three tomato landraces (Ciettaicale, Corleone, and Linosa) and a commercial variety (UC-82B). Error bars represent the standard error of the mean (n = 6). Bars with same letters are not statistically different from one another according to Duncan’s test (P < 0.05).

Landraces treated with 60 mM NaCl did not show changes in lycopene content (**Figure 4**). However, Corleone fruits under 120 mM NaCl contained about 40% less lycopene compared to controls. Notably, UC-82B in control conditions showed the highest values of fruit lycopene among the tomato genotypes, but exhibited a progressive decrease in lycopene content already at 60 mM NaCl (-38%) and more marked at 120 mM NaCl (-55%) compared to the control.

### Total Flavonoids, Total Phenols, and Total Antioxidant Activity

**Figure 5:**
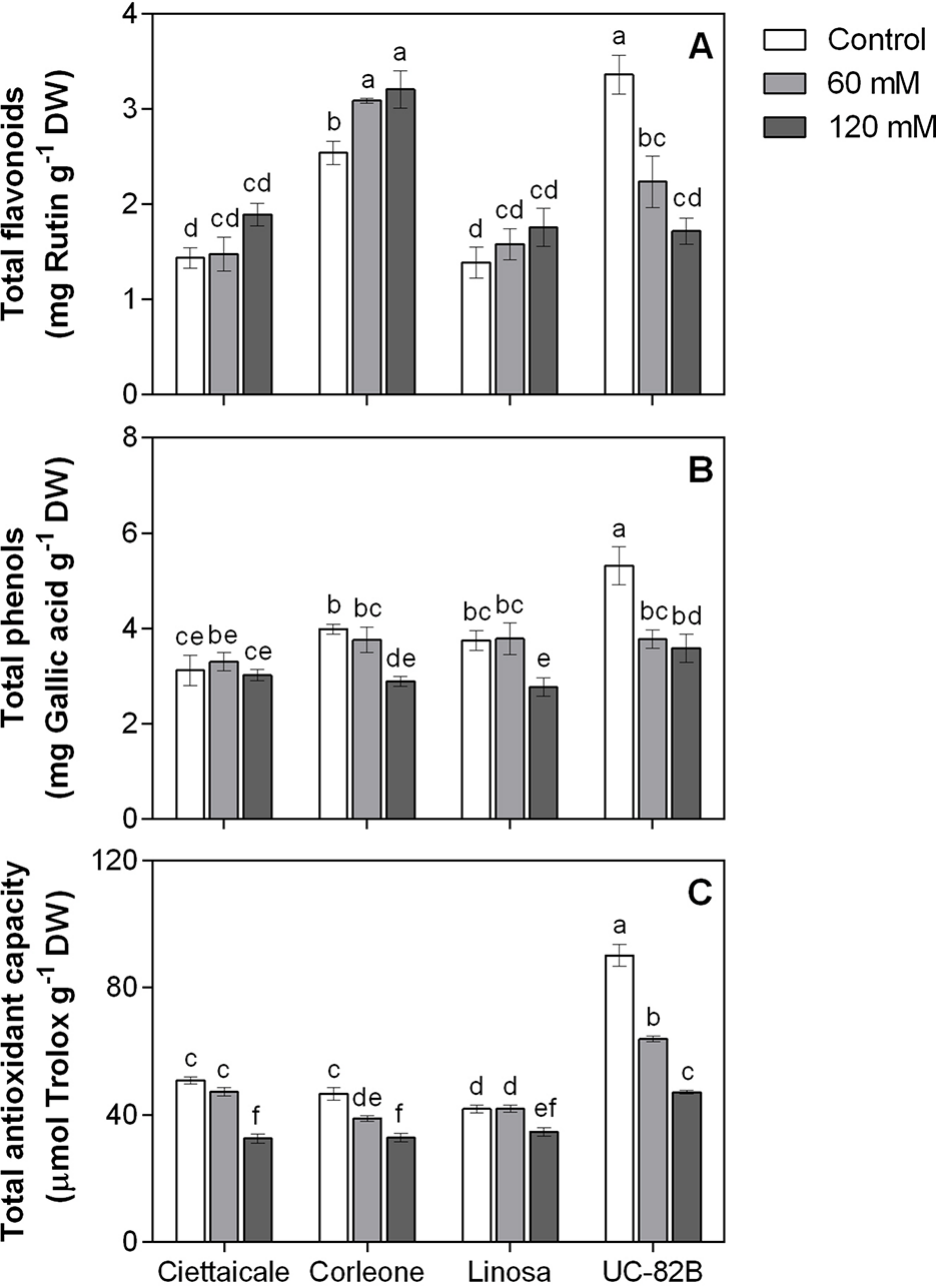
Effect of different salt concentrations (60 mM and 120 mM NaCl) on fruit phenolic content and antioxidant capacity of three tomato landraces (Ciettaicale, Corleone and Linosa) and a commercial variety (UC-82B). **(A)** Total flavonoids, **(B)** total phenols and **(C)** total antioxidant capacity. Error bars represent the standard error of the mean (n = 6). Bars with same letters are not statistically different from one another according to Duncan’s test (P < 0.05).

Salt conditions did not affect TFL in Ciettaicale and Linosa (**Figure 5A**). An increase (+ 21%) in TFL was recorded in Corleone fruits under 120 mM NaCl compared to controls. Conversely, salinity negatively affected TFL in UC-82B. No differences in TPHE in the landraces under 60 mM NaCl were recorded (**Figure 5B**). Under the same stress condition, UC-82B reduced TPHE by around 29% compared to its control. The highest salt concentration caused a decrease in fruit TPHE with a similar magnitude in Corleone, Linosa and UC-82B compared to respective controls (-26%, -28% and -33%, respectively). At harvesting point, fruits of Ciettaicale and Linosa maintained roughly control TAC values under 60 mM NaCl (**Figure 5C**). Corleone and UC-82B had decreased TAC under 60 mM (-17% and -29% compared to their respective controls). However, a common reduction in TAC was observed in all genotypes under 120 mM compared to controls, i.e. from -17% in Linosa to -48% in UC-82B.

### Metabolite Profiling

**Figure 6:**
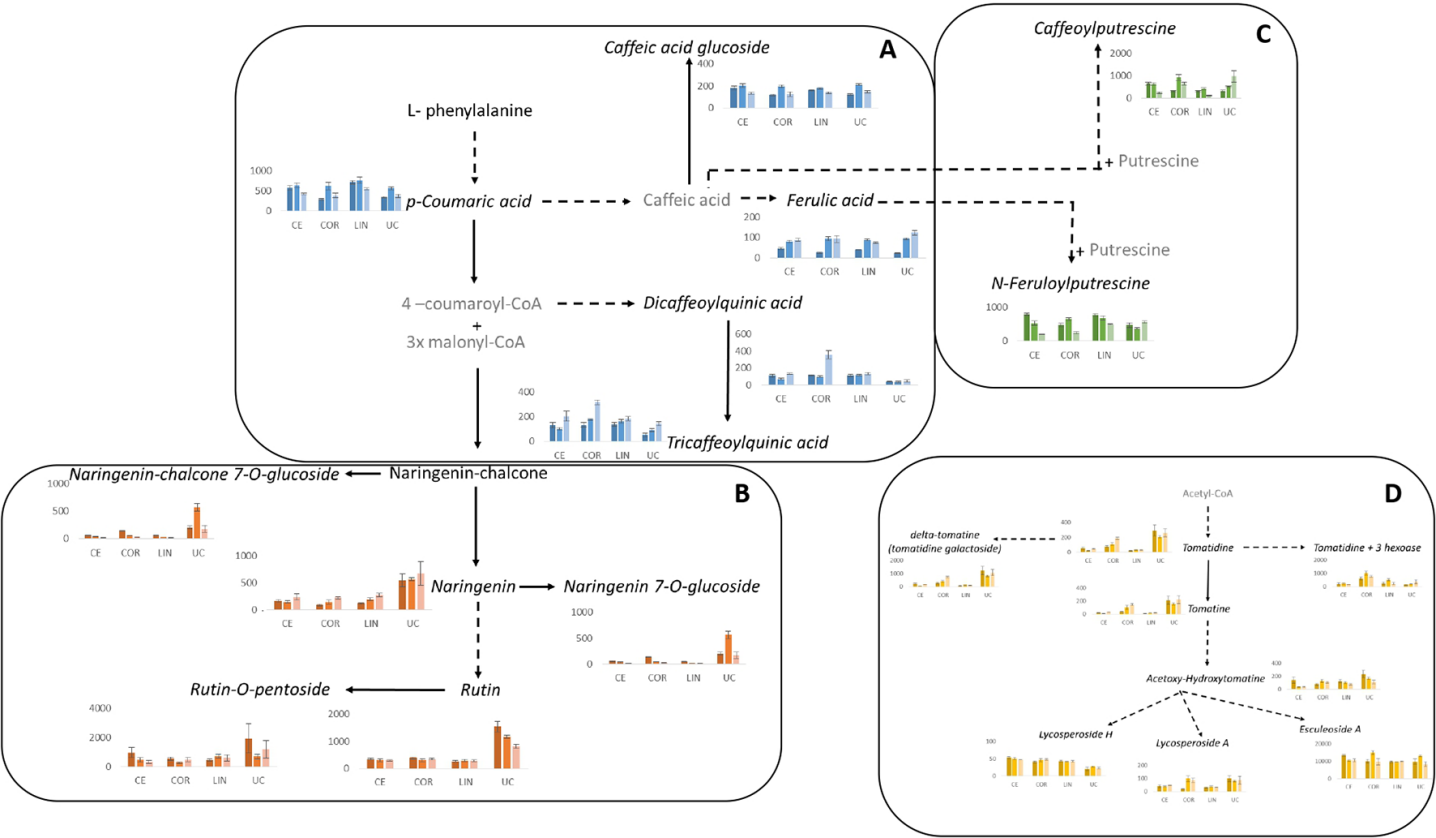
Pathways overview of the plant specialized metabolites responsive to salinity stress. **(A)** Hydroxycinnamic acids, **(B)** flavonoids, **(C)** phenylamides and **(D)** glycoalkaloids. Metabolites that were identified in the present study are represented in black, whereas non identified metabolites are in grey. Metabolites that showed statistically significant changes (genotype x salinity treatment) are represented in italic and graphical representations are presented. The graphs represent the mean of relative ion intensity/dry weight of six biological replicates ± SE. Dark, middle and light colours (blue, orange, green and yellow) represent control, 60 and 120 mM NaCl, respectively; CE, Ciettaicale; LIN, Linosa; COR, Corleone; UC, commercial variety UC-82B.

An untargeted UPLC-MS/MS analysis profiled the same sample sets as described above. We were able to identify 32 metabolites (**Table 3**) based on accurate mass measurements and MS/MS spectra from biological standards or publicly available data, namely literature and/or databases such as PubChem and Moto. ANOVA results are reported in **Supplementary Table 1** and graphically represented in **Figure 6**. Most metabolites detected were phenylpropanoids (10 hydroxycinnamic acids and 7 flavonoids) and glycoalkaloids (8). The remainder metabolites were assigned as phenylamides (4), amino acids (2) and vitamins (1).

**Table 3:** UPLC-MS/MS analysis of plant specialized metabolites responsive to salinity stress found in this work. For the identification level: A, literature; B, database, S, standard.

Peak #	Ret time (min)	Metabolite name	Metabolite class	Code	Molecular formula	[M+H] _ob_	[M+H] _theo_	MS/MS fragments	Identificaton level	Ref
1	3.33	Phenylalanine	Aminoacids	AA1	C_9_H_11_NO_2_	166.0869	166.0863	120, 103	B	PubChem
2	4.52	Tryptophan		AA2	C_11_H_12_N_2_O_2_	205.0976	205.0971	188, 170, 159, 146, 118	B	PubChem
3	7.90	Naringenin chalcone	Flavonoids	FL1	C_15_H_12_O_5_	273.0763	273.0757	153, 147, 119	S, A, B	Moço et al., 2006; MoTo
4	8.05	Naringenin		FL2	C_15_H_12_O_5_	273.0764	273.0757	153, 147, 119	S, A, B	Moço et al., 2006; MoTo
5	6.43	Naringenin 7-O- glucoside		FL3	C_21_H_22_O_10_	435.1304	435.1285	273, 153	A, B	Moço et al., 2006; MoTo
6	6.80	Naringenin chalcone 7-O- glucoside		FL4	C_21_H_22_O_10_	435.1298	435.1285	273, 153	A, B	Moço et al., 2006; MoTo
7	5.81	Phloretin 3’,5’-di-C-glucoside		FL5	C_27_H_34_O_15_	599.1970	599.1990	497, 479, 461, 449, 431, 419, 413, 407, 395, 383, 377, 365, 353, 341, 329, 301, 259, 247, 235, 107	A	Slimestad et al., 2008; Beelders et al., 2014
8	5.69	Rutin		FL6	C_27_H_30_O_16_	611.1616	611.1607	303	S, A, B	Moço et al., 2006; MoTo
9	5.39	Rutin-O-pentoside		FL7	C_32_H_38_O_20_	743.2041	743.2029	465, 303	A, B	Moço et al., 2006; MoTo
10	6.52	Tomatidine	Glycoalkaloids	GA1	C_27_H_45_NO_2_	416.3524	416.3523	398, 273, 255, 161	A, B	Caprioli et al., 2015; MoTo
11	6.50	Delta-tomatine		GA2	C_33_H_55_NO_7_	578.4057	578.4051	417, 273, 255, 161	A, B	Cataldi et al., 2005; PubChem
12	5.95	Tomatidine+3hexoase		GA3	C_45_H_75_NO_18_	918.5081	918.5057	432, 245, 162	A	Iijima et al., 2008
13	6.50	Tomatine		GA4	C_50_H_83_NO_21_	1034.5538	1034.5530		A, B	Moço et al., 2006; MoTo
14	5.72	Lycoperoside H		GA5	C_50_H_83_NO_22_	1050.5475	1050.5480	1032, 594, 432, 325, 273, 255, 163, 145, 127	A	Adato et al., 2009
15	6.45	Lycoperoside A		GA6	C_52_H_85_NO_23_	1092.5577	1092.5585		A	Moço et al., 2006
16	5.93	Acetoxy-Hydroxytomatine		GA7	C_52_H_85_NO_24_	1108.5517	1108.5534		A	Iijima et al., 2008
17	5.55	Esculeoside A		GA8	C_58_H_95_NO_29_	1270.6011	1270.6063	1210, 1090, 1048, 1030, 652, 592	A	Iijima et al., 2008
18	4.33	Coumaric acid I	Hydroxycinnamic acids	HC1	C_9_H_8_O_3_	165.0550	165.0546	147, 119, 91	A, B	Moço et al., 2006; MoTo
19	4.99	Coumaric acid II		HC2	C_9_H_8_O_3_	165.0552	165.0546	147, 119, 91	A, B	Moço et al., 2006; MoTo
20	4.59	Ferulic acid I		HC3	C_10_H_10_O_4_	195.0659	195.0651	177, 145, 117	A, B	Moço et al., 2006; MoTo
21	6.25	Ferulic acid II		HC4	C_10_H_10_O_4_	195.0663	195.0651	177, 145, 117	A, B	Moço et al., 2006; MoTo
22	4.33	Coumaric acid-hexose		HC5	C_15_H_18_O_8_	327.1082	327.1074	165, 147, 119	A, B	Moço et al., 2006; MoTo
23	4.28	Caffeic acid glucoside I		HC6	C_15_H_18_O_9_	343.1035	343.1023	181, 163	A, B	Moço et al., 2006; MoTo
24	4.80	Caffeic acid glucoside II		HC7	C_15_H_18_O_9_	343.1033	343.1023		A, B	Moço et al., 2006; MoTo
25	6.40	1,3-O-Dicaffeoylquinic acid I		HC8	C_25_H_24_O_12_	517.1351	517.1341	163	B	PubChem
26	6.77	1,3-O-Dicaffeoylquinic acid II		HC9	C_25_H_24_O_12_	517.1354	517.1341	323, 295, 273, 163	B	PubChem
27	7.15	3,4,5-Tricaffeoylquinic acid		HC10	C_34_H_30_O_15_	679.1679	679.1657	499, 163		
28	3.24	Caffeoylputrescine I	Phenylamides	PA1	C_13_H_18_N_2_O_3_	251.1399	251.1390	234, 163, 145, 135,117	A	Gaquerel et al., 2010
29	3.96	Caffeoylputrescine II		PA2	C_13_H_18_N_2_O_3_	251.1398	251.1390	234, 163, 145, 135,117	A	Gaquerel et al., 2010
30	4.36	Feruloylputrescine I		PA3	C_14_H_20_N_2_O_3_	265.1539	265.1546	177, 145, 117	B	PubChem
31	4.60	Feruloylputrescine II		PA4	C_14_H_20_N_2_O_3_	265.1532	265.1546	177, 145, 117	B	PubChem
32	3.92	Pantothenic acid-hexose	Vitamins	VA1	C_15_H_27_NO_10_	382.1722	382.1707	252, 220, 202, 184, 116, 90	A	Mintz-Oron et al., 2008

Among the hydroxycinnamic acids (**Figure 6A**), coumaric acid (detected as two isomers) was the compound that presented the most intense mass signal (**Supplementary Table 1**). We could observe only a significant decrease of coumaric acid II under 120 mM NaCl in UC-82B. Interestingly, a similar trend was observed for coumaric acid-hexose accumulation in Linosa. Two ferulic acid isomers were detected: ferulic acid I significantly increased in all genotypes under salt stress compared to control conditions, whereas ferulic acid II did not significantly change in the case of Ciettaicale and Corleone upon salt stress. Linosa and UC-82B displayed a similar trend of reduction of the intensity of ferulic acid II mass signal upon 120 mM NaCl. The 1,3-O-dicaffeoylquinic acid I showed a remarkably low mass signal intensity in UC-82B fruits as compared to the landraces. At 120 mM NaCl, Corleone fruits showed a 3 fold increase of 1,3-O-dicaffeoylquinic acid I mass signal intensity. Also, 3,4,5-tricaffeoylquinic acid showed a significant increase in mass signal intensity in Corleone fruits upon 120 mM NaCl.

Apart from hydroxycinnamic acids, the phenylamides conjugated with caffeic acid (caffeoylputrescine I and II isomers) and ferulic acid (feruloylputrescine I and II isomers) were also identified (**Figure 6C** and **Supplementary Table 1**). Caffeoylputrescine I was significantly reduced in Ciettaicale and Linosa upon 120 mM NaCl, whereas the same salt concentration induced an increase of this compound in UC-82B fruits. Feruloylputrescine I was significantly reduced under 120 mM NaCl for the three landraces. Feruloylputrescine II increased in Corleone and UC-82B fruits upon salt stress, but in Linosa fruits the opposite trend was observed.

Regarding the flavonoids identified, rutin was the compound that exhibited the most intense mass signal (**Figure 6B** and **Supplementary Table 1**). Interestingly, UC-82B fruits showed the highest signal for all the flavonoids detected. Nevertheless, rutin and rutin-O-pentoside did not significantly change upon salt stress in all landraces, whereas UC-82B fruits showed a significant decrease of rutin upon 60 and 120 mM NaCl.

The list of identified compounds also includes 8 glycoalkaloids that presented a variable accumulation pattern in the studied conditions (**Figure 6D** and **Supplementary Table 1**). Esculeoside A was the glycoalkaloid that showed the highest mass signal intensity. Under control conditions, Ciettaicale accumulated significantly higher amounts of esculeoside A than all the other genotypes, while 60 mM NaCl promoted its accumulation in Corleone and UC-82B fruits. Under 120 mM NaCl all genotypes accumulated similar levels of esculeoside A. Tomatidine content was significantly higher in the commercial variety and Corleone under 120 mM NaCl, when compared to Ciettaicale and Linosa. Tomatidine+3hexoase showed the highest mass intensity signal in Corleone fruits upon 60 mM NaCl treatment. Tomatine, delta-tomatine and lycosperoside A were significantly high in the commercial variety in all conditions and in Corleone upon 120 mM NaCl. On the other hand, lycoperoside H was significantly higher in all landraces compared to UC-82B variety, and its content was not modulated by salt treatment.

We also identified the amino acids phenylalanine and tryptophan, and the vitamin derivative, pantothenic acid–hexose (**Supplementary Table 1**). Phenylalanine showed a marked increase in mass signal intensity upon 60 mM NaCl treatment in Ciettaicale fruits, whereas for the other genotypes the levels remained unchanged. The lowest mass signal intensity of tryptophan was observed in Corleone at 120 mM NaCl treatment, whereas the other genotypes showed the same mass signal intensity for this compound. Finally, the levels of pantothenic acid-hexose were high upon 60 mM NaCl in all genotypes except for Linosa, which showed unaltered levels of this metabolite in all treatments.

Moreover, the UPLC-MS/MS data were used in a further analysis known as SIMPER (**Supplementary Table 2**). The objective of this analysis was to find key compounds that allow for differentiation of one experimental condition from another when compared in a pair-wise analysis. The results display the contribution of each compound to the average overall dissimilarity of the two compared samples. A cut-off is imposed when ∑δi% reaches 70%. The metabolomic profiles of the experimental treatments differed qualitatively. In the first analyses, we identified the compounds that were consistently present in all the three salinity comparisons (control vs 60, control vs 120, and 60 vs 120), regardless of the genotypes. Among them, comparing conspecific different salinity treatments among all the genotypes, we identified the persistence of seven compounds: feruloylputrescine II (PA4), caffeoylputrescine I (PA1), naringenin (FL2), rutin-O-pentoside (FL7), naringenin chalcone (FL1), esculeoside A (GA8) and tomatidine+3hexoase (GA3).

**Figure 7:**
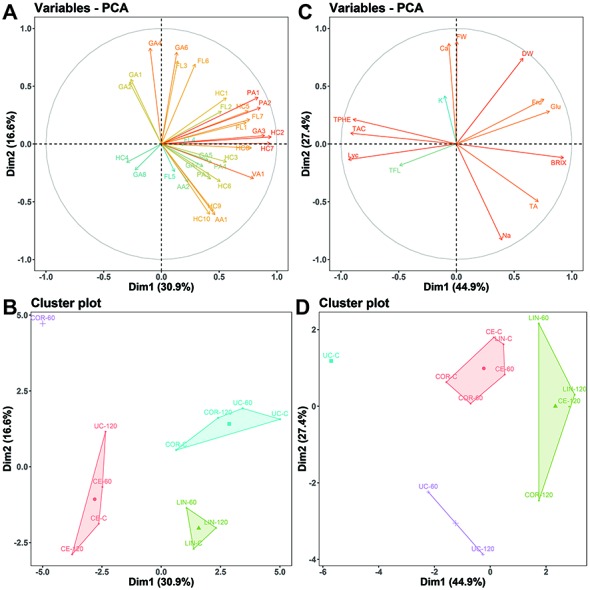
Graphical representations of Principal Component Analysis (PCA) results. **(A** and **C)** Vector representation of the contribution of each metabolite identified by UPLC-MS/MS **(A)** and each laboratory determination/assay **(C)** to the distinction of experimental treatments. The coordinates of each variable are the correlation coefficients with the two-first principal components. Between vectors and between a vector and an axis, there is positive correlation if the angle is less than 90 degrees whereas the correlation is negative if the angle reaches 180 degrees. There is no linear dependence if the angle is 90 degrees. **(B** and **D)** Cluster map of PCA results of UPLC-MS/MS data **(B)** and laboratory determinations/assays **(D)** obtained from experimental treatments. For UPLC-MS/MS metabolite codes see **Table 3**; FW, yield fresh weight; DW, yield dry weight; Na, sodium; K, potassium; Ca, calcium; Fru, fructose; Glu, glucose; BRIX, total soluble solids; TA, titratable acidity; LYC, lycopene; TPHE, total phenols; TFL, total flavonoids; TAC, total antioxidant capacity; CE, Ciettaicale; COR, Corleone; LIN, Linosa; UC, commercial variety UC-82B; C, control condition; 60 and 120 mM NaCl used as experimental treatments, respectively.

The PCA (**Figures 7A, B**) performed on metabolomics data showed that the first two dimensions (PC1 and PC2) account for 47.5% of the total variance (total inertia). The first axis (PC1) explains 30.9% of the total variance and the second axis (PC2) 16.6%. The contribution of individual compounds to sample differentiation is displayed as a correlation circle (**Figure 7A**) where normalized vectors graphically represent the quantitative variables. The length and the direction of the vectors directly correlate with their significance within each treatment. A positive correlation between compounds is greater when the angle between their directions is smaller (close to 0 degree), whereas the correlation is negative if the angle reaches 180 degrees. No linear dependence exists if the angle is exactly 90 degrees. Overall, in our dataset we observed strong positive correlation between caffeic acid glucoside II (HC7) and coumaric acid II (HC2), caffeoylputrescine I (PA1) and caffeoylputrescine II (PA2), and 1,3-O-dicaffeoylquinic acid II (HC9), 3,4,5-tricaffeoylquinic acid (HC10) and phenylalanine (AA1). From the spatial distribution of the treatments (**Figure 7B**), we observed that Corleone distinguished itself the most. At 60 mM NaCl, Corleone showed a distinct metabolomic profile, while it shared more similarities under control conditions and at 120 mM NaCl. Ciettaicale and Linosa clustered separately but formed one cluster regardless of the salinity level. Furthermore, the commercial variety UC-82B under 120 mM NaCl showed a distinct profile compared to the control and mild salinity level. In the PCA analysis performed on the biochemical and biometric characteristics (**Figures 7C, D**), the landraces segregated into two main groups mainly according to the salinity level, whereas the standard variety clustered separately. In particular, untreated UC-82B was uniquely distinct, while the salt-treated ones grouped together forming one cluster.

## Discussion

Tomato landraces are a valuable resource for many traits of agronomic interest. This is mostly due to their resilience against abiotic stresses, which contributes to yield stability and adaptation to low input and/or adverse growth conditions. Landraces are also associated with distinctive organoleptic and nutritional quality traits and could exhibit peculiar and often contrasting metabolic profiles (Baldina et al., 2016; Gascuel et al., 2017; Patanè et al., 2017; Siracusa et al., 2018). High contents in functional compounds are frequent traits found in Mediterranean traditional tomato varieties (Pinela et al., 2012; Berni et al., 2018), but often no differences in sensory profile have been identified between commercial varieties and landraces (Ruiz et al., 2005; Sinesio et al., 2007; Casals et al., 2011). Moreover, the promoting effect on fruit quality metabolites is frequently ascribed to the concentration effect and not to the absolute accumulation (Zushi and Matsuzoe, 2015). However, different studies consistently concluded that the interaction between genotype and environment is the key component able to modulate the expression of specific metabolic patterns (Berni et al., 2019).

In this study we compared the effects of moderate and high concentrations of sodium chloride on yield and quality-related ions and metabolites in three tomato landraces (Ciettaicale, Corleone and Linosa) and a commercial variety (UC-82B). We showed that salinity promoted the anticipation of fruit ripening in all genotypes, but differentially caused fresh fruit yield losses. Notably, at 60 mM NaCl all landraces showed better performance in terms of yield FW compared to the commercial variety. The most interesting results were the absence of yield loss in Corleone and Linosa at the aforementioned salinity level and the observation that Linosa reduced only around 20% fresh fruit production under 120 mM NaCl. The capacity to maintain an adequate yield has also been found in a Kenyan tomato landrace grown in site soil polluted by high salty water irrigation (Agong et al., 1997).

Yield and functional quality traits can be influenced by salinity mainly due to sodium competition for other cations, such as K^+^ and Ca^2+^ (Adams, 1991; Petersen et al., 1998, Pompeiano et al., 2017). Among the genotypes, Linosa maintained higher K^+^/Na^+^ and Ca^2+^/Na^+^ ratios along the salt gradient. Calcium participates both in the alleviation of sodium toxicity and in the fruit size development (Plieth, 2005; Manaa et al., 2013). This observation could support the ability of Linosa to maintain an adequate yield under salt stress. Additionally, fresh tomato fruits with high Ca^2+^ content represent a natural mineral supply indispensable in human dietary (Soetan et al., 2010). On the contrary, in the commercial variety the Ca^2+^/Na^+^ ratio was more affected by the salinity gradient, leading to a more marked reduction of fruit size and weight. Several studies reported that calcium deficiency affects tomato fruit development (Park et al., 2005), often resulting with the appearance of the blossom-end rot (Taylor and Locascio, 2004). However, in the present study, as well as in the winter greenhouse experiment conducted by Zushi and Matsuzoe (2009), the fruits of all genotypes were not affected by this marketable injury.

The increases in soluble solids and titratable acidity are common responses of tomato fruits under salt stress (Balibrea et al., 2003; Zushi and Matsuzoe, 2015). High values of these parameters have been found in Spanish tomato landraces under salt stress (Massaretto et al., 2018). Soluble solids content (°Brix) mainly estimated the sugar amounts in tomato fruit pulp, but also organic acids, amino acids, soluble pectins, phenolic compounds and minerals (Beckles, 2012). Nevertheless, the sugar/acid ratio generally increases during summer and decreases during winter. Primary metabolism is more affected when light and temperature changes occur during early fruit development than when environmental conditions mutate during the ripening phase (Gautier et al., 2008). Under 120 mM NaCl all genotypes showed higher °Brix content compared to the respective controls. High °Brix improves the taste of tomato fruits and is a desirable trait for the processing of tomato products (De Pascale et al., 2001). Also 120 mM NaCl promoted the accumulation of fructose and glucose in the landraces, but not in the commercial variety. Zushi and Matsuzoe (2015) reported that the tomato cultivar Mini Carol accumulated more fruit glucose and fructose under 50 mM NaCl, while the tomato cultivar House Momotaro increased total soluble sugars only under 100 mM NaCl. The authors concluded that the salt effect on sugar levels depends essentially on the genotype. Interestingly, sucrose was detectable only in traces in the fruits of any of the studied tomato accessions, suggesting that salt stress promotes invertase activity and consequently the release of hexoses during tomato ripening (Balibrea et al., 2003). Also, low solar radiation conditions, such as the ones experienced in our study, could affect sugar concentration in sink tissues due to a limitation in carbon fixation/transport in/from source leaves (Hu et al., 2006).

The content of lycopene, the main carotenoid that confers the red pigmentation to the tomato fruit, is a genotype-dependent trait. Lycopene metabolism can be modulated by water deficit (Atkinson et al., 2011; Coyago-Cruz et al., 2017), low light radiation as well as low temperature (Dumas et al., 2003; Jarquín-Enríquez et al., 2013). Even though several tomato landraces have been identified with constitutive high carotenoid content (Pinela et al., 2012; Figàs et al., 2015; Zushi and Matsuzoe, 2015), the studied landraces showed lower fruit lycopene content under control conditions compared to the commercial variety. Upon 60 mM NaCl treatment, the landraces roughly maintained control values of lycopene, while the commercial variety had a significantly reduced content. Ciettaicale and Linosa displayed no changes in lycopene amount under 120 mM NaCl. The decrease in K^+^ content, as we observed in Corleone and UC-82B under 120 mM NaCl, could affect lycopene production. Indeed, K^+^ plays a role as cofactor of several enzymes involved in the biosynthesis of isopentenyl diphosphate, the first precursor of carotenoids in the mevalonate pathway (Trudel and Ozbun, 1971). Li and Yuan (2013) and Coyago-Cruz et al. (2019) suggested that the fruit ripening-related accumulation of lycopene can also be influenced due to limited amount of sucrose. Overall, our results were in agreement with those found by Petersen et al. (1998), which reported that salinity did not promote an increase of lycopene levels per dry weight in tomato fruits. Nevertheless, moderate salinity stress has previously been used to improve lycopene content in tomato (De Pascale et al., 2001; Kubota et al., 2012).

Phenolic acids and flavonoids represent a complex class of compounds with specific biological activity. Their profile in tomato fruits have been widely investigated and often used as taxonomical markers to discriminate tomato varieties (Minoggio et al., 2003; Vallverdú-Queralt et al., 2011). Flavonoids content has been positively correlated with environmental radiation, resulting in seasonal changes (Incerti et al., 2009). For example, one light-dependent effect is the up-regulation of the gene expression of chalcone synthase, the first committed enzyme in flavonoid biosynthesis (Feinbaum and Ausubel, 1988). Flavonoids have been found highly concentrated in epidermal and placental tissues of tomato fruits, acting as chemical defences against pathogens and UV radiation (Slimestad et al., 2008). Flavonoids, also known as vitamin P, have recently been targeted as important functional compounds with benefits for human health (Perez-Vizcaino and Fraga, 2018). New emerging studies showed that flavonoids may interfere with the signalling of several kinases that in turn modulate cellular functions by altering the phosphorylation state of target molecules and by modulating gene expression (Williams et al., 2004; Van Der Rest et al., 2006).

Most of the flavonoids found in tomato are mainly present as O-glycosides, but also as non-conjugated forms (aglycones). The main flavonoid reported in tomato fruits is rutin (quercetin 3-rutinoside) (Le Gall et al., 2003). However, naringenin chalcone and its more stable cyclized form naringenin, as well as naringenin-7-O-glucoside, have been also detected as additional frequent flavonoids in fresh tomato skins (Moço et al., 2006) and have been investigated *in vitro* as potential anti-allergic compounds (Yamamoto et al., 2004). In addition, the presence of the aglycones kaempferol, quercetin and naringenin is also noticed, but with a discrepancy in the amount among experimental studies (Stewart et al., 2000). Overall, the content of flavonoids in terms of quantity and quality greatly varies depending on genotype, growth conditions, stage of ripeness and tissue, as well as on the detection method (Slimestad and Verheul, 2005; Slimestad et al., 2008). In this study, most of the abovementioned flavonoids were identified with the exception of the aglycones, possibly due to the absence of acid hydrolysis of the analysed sample extracts. The commercial variety clearly differed from the landraces for rutin and rutin-O-pentoside contents both under control and salt. Overall, the TFL content, as well as rutin content, in the commercial variety decreased according to the salt gradient. In Ciettaicale and Linosa, TFL content was not affected by salinity, while 120 mM NaCl induced an accumulation of TFL in Corleone fruits.

Tomato fruits also contain hydroxycinnamic acids and respective quinic acid ester derivatives. During ripening, an increase of these esters generally occurs, especially in the pulp (Whitaker and Stommel, 2003). Caffeic acid and its quinic acid ester (chlorogenic acid), as well as ferulic acid and coumaric acid, are detected in quite high levels in tomato fruits. In particular, chlorogenic acid and its derivatives reduce the incidence of fungal disease in tomato (Ruelas et al., 2006; Wojciechowska et al., 2014). Moreover, hydroxycinnamic acids can modulate auxin and ethylene metabolism, which are both involved in fruit size development and ripening (Fleuriet and Macheix, 1981). The highest salt stress condition promoted di- and tricaffeoylquinic acid accumulation in Corleone compared to the commercial variety. Coumaric acid (I+II) showed a similar profile of accumulation in all genotypes under 60 mM NaCl, while a decreasing trend was observed in Linosa and in the commercial variety under 120 mM NaCl compared to the respective controls. Cinnamate 3-hydroxylase, a key limiting enzyme for hydroxycinnamic acid biosynthesis (Ferrer et al., 2008), was shown to be up-regulated at transcriptional and protein levels by salinity (Martinez et al., 2016). Our data suggest that cinnamate 3-hydroxylase may be differentially regulated in the landraces compared to the commercial variety, but further investigations need to be conducted to validate this hypothesis.

The observed decrease in TPHE in salt-treated plants may be the result of the reallocation of phenolic compounds to lignin polymers as a protective mechanism (Humphreys and Chapple, 2002). However, decreased values of phenolic contents are often observed under low temperature (Rivero et al., 2001; Gautier et al., 2008).

Tomato, a fully-fledged member of the *Solanaceae*, produces steroidal glycoalkaloids, which belong to the terpenoid family. Tomatine represents the main glycoalkaloid in tomato fruit. From a pharmaceutical point of view, dietary tomatine leads to a reduction of plasma cholesterol content thanks to the capacity of this terpenoid to form insoluble complexes with cholesterol, which are poorly absorbed from the intestinal tract (Friedman, 2013). During fruit ripening, tomatine is normally converted in esculeoside, while concomitantly lycopene contents increases (Katsumata et al., 2011). However, as for many other metabolites, this relationship is deeply affected by agronomic practices, genotype and environmental conditions (Koh et al., 2013). The pattern of accumulation of glycoalkaloids was variable in the landraces and in the commercial variety upon salt stress. The levels of esculeoside A were almost similar in all genotypes. Although salinity was shown to induce accumulation of these compounds in tomato leaves (Han et al., 2016), this trend was only confirmed for Corleone.

The class of polyamines, which includes putrescine, spermidine and spermine, is required for tomato fruit development (Cohen et al., 1982). These metabolites can donate amino groups to different other plant compounds, such as hydroxycinnamic acids. Hydroxycinnamic amides seem to play a key role in the reproductive tissues since their catabolism provides nitrogenous and phenolic carbon skeletons for reproductive development (Balint et al., 1987). Caffeoylputrescine and feruloylputrescine isomer contents showed different trends in the genotypes along the salt gradient. Since polyamines compete with ethylene for the biosynthetic precursor S-adenosylmethionine, high content of hydroxycinnamic amides may delay the fruit softening-inhibiting production of ethylene (Liu et al., 2006).

Overall, despite the salinity-induced rearrangement in the stoichiometry of the antioxidant metabolites identified by UPLC-MS/MS, UC-82B and Corleone progressively decreased fruit TAC with increasing salt concentrations, while in Ciettaicale and Linosa TAC only declined under 120 mM NaCl.

## Conclusion

The combination of moderate/high salt concentrations with low light irradiance differently affected the yield and the metabolism of the studied tomato genotypes. Despite these non-optimal environmental conditions for tomato cultivation, the Italian landraces showed a different behaviour as compared to the commercial variety UC-82B under moderate salinity stress, showing a tolerable compromise between yield and quality attributes. Salt stress markedly reduced yield and functional metabolite contents in the commercial variety. Among the landraces investigated, Linosa showed better performance in terms of yield/quality parameters under 60 mM NaCl. However, off-season high salinity stress (120 mM NaCl) significantly reduced the antioxidant activity both in UC-82B and in the landraces. In conclusion, these data point to the use of tomato landrace germplasm as a suitable strategy to counteract detrimental environmental factors, such as salinity and off-season cropping, and also as resource of metabolic biomarkers which can be used to improve commercial varieties.

## Data Availability

All datasets for this study are included in the manuscript and the **Supplementary files**.

## Author Contributions

TM, RBF, LM, LI, and FS conceived and designed the experiments with the help of all authors. TM, RBF, LM, GC, AS, and LP performed the experiments. TM, RBF, AnP, AL, and LI analyzed the data. TM, RBF, and AnP drafted the manuscript. All authors led the critical review of the final version.

## Funding

This research has been financially supported by the Swiss National Science Foundation (grant no. 31003A-166539/1 to DS). AnP was supported by the project no. LQ1605 from the National Program of Sustainability II (MEYS CR).

## Conflict of Interest Statement

The authors declare that the research was conducted in the absence of any commercial or financial relationships that could be construed as a potential conflict of interest.
