# Supplementary material for: Salinity in Autumn-Winter Season and Fruit Quality of Tomato Landraces
Source: Front Plant Sci. 2019 Sep 24;10:1078. doi: 10.3389/fpls.2019.01078 (PMC6769068; doi:10.3389/fpls.2019.01078)
Supplement: Supplementary file 2 [file Table_2.docx]

**Supplementary Table 2.** SIMPER results of pair-wise comparisons on selected samples. **(A)** Representation of the pair-wise comparison Control vs. 120 mM NaCl; **(B)** Representation of the pair-wise comparison Control vs 60 mM NaCl; **(C)** Representation of the pair-wise comparison 60 mM NaCl vs 120 mM NaCl. Compound codes as reported in Table 3. The compounds are listed in the order of their contribution (δ_i_) to the average dissimilarity between the two groups, with a cut-off when the cumulative percent contribution (∑δ_i_%) to δ_i_ reaches 70%. Red mark indicates common compounds within each pair-wise comparison; asterisk (*) indicates common compounds with all the pair-wise comparisons.

**(A)**

| CE-C_CE-120 | | COR-C_COR-120 | | LIN-C_LIN-120 | | UC-C_UC-120 | |
| --- | --- | --- | --- | --- | --- | --- | --- |
| Code | ∑δ_i_% | Code | ∑δ_i_% | Code | ∑δ_i_% | Code | ∑δ_i_% |
| FL1* | 0.056 | GA8 | 0.064 | FL1 | 0.077 | FL2 | 0.057 |
| FL2* | 0.109 | FL2 | 0.115 | FL2 | 0.133 | PA1 | 0.113 |
| AA1 | 0.161 | PA3 | 0.165 | GA3 | 0.189 | AA1 | 0.166 |
| GA8* | 0.209 | FL6 | 0.213 | PA4 | 0.239 | GA3 | 0.218 |
| GA3* | 0.250 | GA3 | 0.259 | FL7 | 0.286 | PA4 | 0.266 |
| PA1* | 0.291 | AA2 | 0.304 | HC1 | 0.331 | FL1 | 0.312 |
| HC10 | 0.331 | FL5 | 0.348 | PA3 | 0.372 | FL7 | 0.356 |
| PA2 | 0.371 | PA2 | 0.389 | FL5 | 0.413 | PA3 | 0.398 |
| PA3 | 0.410 | FL3 | 0.428 | AA1 | 0.454 | HC1 | 0.439 |
| FL7* | 0.448 | GA2 | 0.467 | PA1 | 0.495 | GA8 | 0.479 |
| HC2 | 0.486 | FL1 | 0.503 | AA2 | 0.532 | FL4 | 0.517 |
| HC8 | 0.522 | HC8 | 0.537 | GA8 | 0.568 | HC2 | 0.553 |
| GA7 | 0.557 | HC2 | 0.572 | HC2 | 0.600 | HC5 | 0.587 |
| HC7 | 0.592 | AA1 | 0.606 | HC10 | 0.632 | AA2 | 0.621 |
| FL6 | 0.624 | PA4 | 0.637 | FL6 | 0.664 | PA2 | 0.654 |
| PA4* | 0.656 | HC10 | 0.667 | HC5 | 0.694 | HC3 | 0.685 |
| VA1 | 0.688 | PA1 | 0.695 | HC3 | 0.721 | GA2 | 0.715 |
| FL5 | 0.719 | FL7 | 0.723 |  |  |  |  |

**(B)**

| CE-C_CE-60 | | COR-C_COR-60 | | LIN-C_LIN-60 | | UC-C_UC-60 | |
| --- | --- | --- | --- | --- | --- | --- | --- |
| Code | ∑δ_i_% | Code | ∑δ_i_% | Code | ∑δ_i_% | Code | ∑δ_i_% |
| AA1 | 0.068 | FL2 | 0.057 | FL1 | 0.070 | FL4 | 0.073 |
| GA8* | 0.128 | PA1 | 0.112 | GA3 | 0.134 | FL1 | 0.145 |
| FL2* | 0.177 | GA8 | 0.164 | PA1 | 0.186 | AA1 | 0.210 |
| PA1* | 0.223 | FL6 | 0.215 | PA3 | 0.237 | PA1 | 0.268 |
| AA2 | 0.267 | HC1 | 0.264 | FL2 | 0.286 | GA8 | 0.325 |
| PA3 | 0.309 | FL1 | 0.309 | PA4 | 0.325 | FL2 | 0.380 |
| FL1* | 0.350 | HC5 | 0.351 | GA8 | 0.364 | FL3 | 0.431 |
| GA3* | 0.389 | PA2 | 0.392 | AA1 | 0.403 | HC1 | 0.478 |
| HC2 | 0.425 | FL3 | 0.430 | FL5 | 0.440 | PA4 | 0.520 |
| PA2 | 0.461 | HC7 | 0.468 | HC1 | 0.478 | FL5 | 0.560 |
| PA4* | 0.497 | HC8 | 0.507 | HC2 | 0.514 | FL7 | 0.593 |
| FL6 | 0.531 | FL7 | 0.545 | FL7 | 0.549 | HC5 | 0.626 |
| HC10 | 0.563 | HC2 | 0.582 | AA2 | 0.583 | GA3 | 0.655 |
| HC1 | 0.595 | AA2 | 0.618 | FL6 | 0.616 | HC6 | 0.683 |
| GA2 | 0.626 | GA3 | 0.653 | HC5 | 0.648 | FL6 | 0.711 |
| HC8 | 0.658 | PA4 | 0.683 | HC6 | 0.679 |  |  |
| HC7 | 0.689 | GA2 | 0.712 | HC10 | 0.709 |  |  |
| FL7* | 0.719 |  |  |  |  |  |  |

**(C)**

| CE-60_CE-120 | | COR-60_COR-120 | | LIN-60_LIN-120 | | UC-60_UC-120 | |
| --- | --- | --- | --- | --- | --- | --- | --- |
| Code | ∑δ_i_% | Code | ∑δ_i_% | Code | ∑δ_i_% | Code | ∑δ_i_% |
| AA1 | 0.071 | PA1 | 0.057 | FL2 | 0.063 | FL2 | 0.056 |
| GA8* | 0.126 | PA2 | 0.112 | PA1 | 0.124 | FL4 | 0.107 |
| FL1* | 0.175 | FL2 | 0.165 | GA3 | 0.184 | GA3 | 0.157 |
| PA1* | 0.223 | GA8 | 0.214 | FL1 | 0.237 | PA1 | 0.205 |
| PA2 | 0.269 | FL1 | 0.262 | PA3 | 0.290 | FL1 | 0.252 |
| HC2 | 0.314 | HC1 | 0.310 | PA4 | 0.343 | FL3 | 0.295 |
| PA3 | 0.358 | HC2 | 0.355 | HC1 | 0.389 | GA8 | 0.337 |
| AA2 | 0.400 | HC8 | 0.401 | AA1 | 0.430 | PA3 | 0.379 |
| FL2* | 0.441 | GA3 | 0.445 | GA8 | 0.470 | AA1 | 0.420 |
| GA3* | 0.482 | FL6 | 0.489 | AA2 | 0.505 | HC1 | 0.461 |
| HC10 | 0.523 | HC5 | 0.532 | HC4 | 0.539 | PA4 | 0.502 |
| HC7 | 0.563 | HC7 | 0.571 | HC2 | 0.571 | FL7 | 0.542 |
| FL7* | 0.602 | FL7 | 0.609 | FL6 | 0.604 | AA2 | 0.580 |
| VA1 | 0.640 | PA3 | 0.645 | FL7 | 0.635 | HC2 | 0.614 |
| HC8 | 0.676 | PA4 | 0.679 | HC10 | 0.665 | HC5 | 0.646 |
| PA4* | 0.708 | GA2 | 0.711 | HC8 | 0.695 | PA2 | 0.677 |
|  |  |  |  | HC5 | 0.724 | FL6 | 0.708 |
