# Supplementary figures and images for: Salinity in Autumn-Winter Season and Fruit Quality of Tomato Landraces
Source: Front Plant Sci. 2019 Sep 24;10:1078. doi: 10.3389/fpls.2019.01078 (PMC6769068; doi:10.3389/fpls.2019.01078)

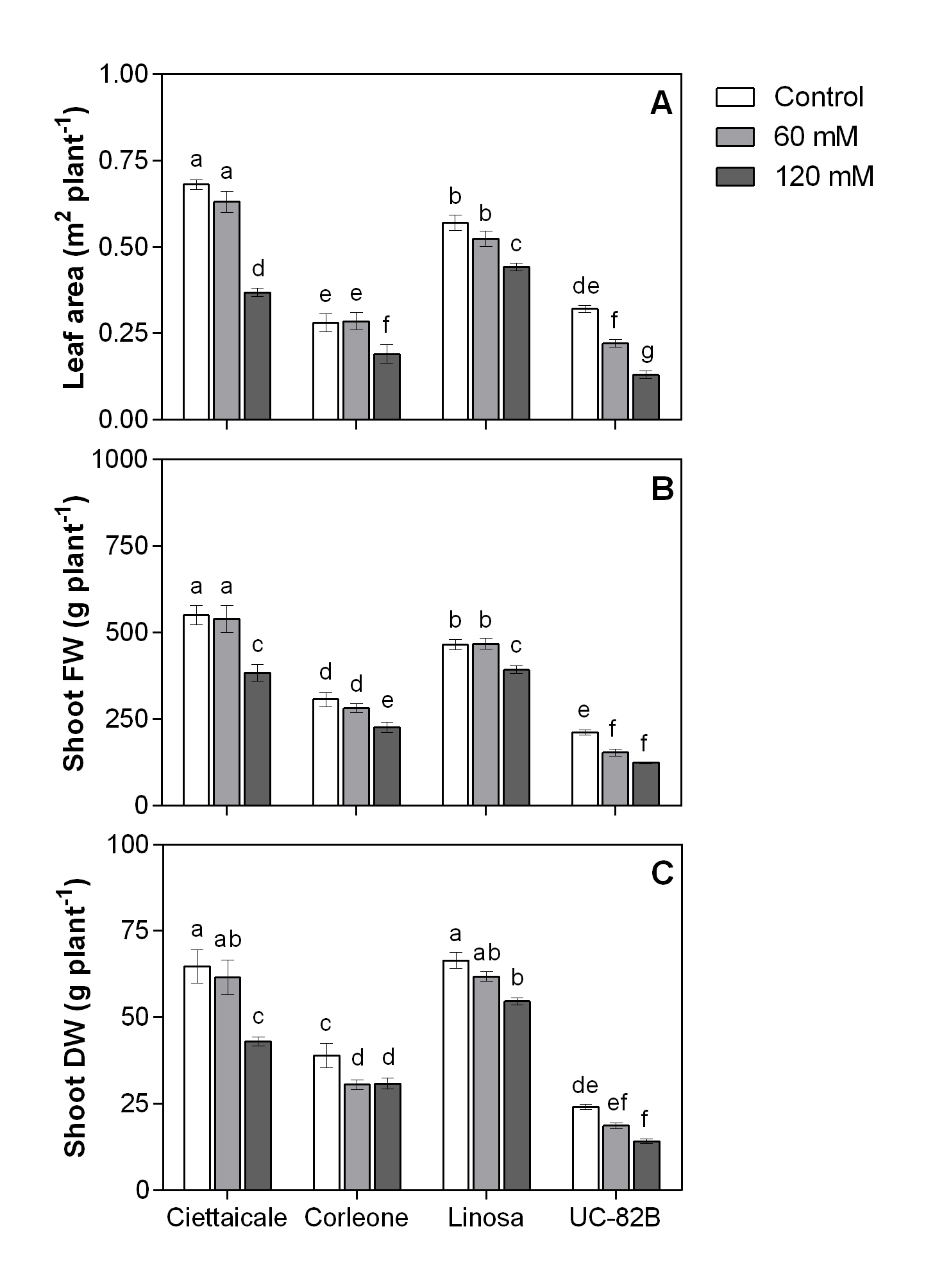

Supplement: Supplementary file 3 [file Image_1.jpeg]
